# Detection and functional characterization of sigma class GST in *Phlebotomus argentipes* and its role in stress tolerance and DDT resistance

**DOI:** 10.1038/s41598-019-56209-0

**Published:** 2019-12-23

**Authors:** Faizan Hassan, Krishn Pratap Singh, Vahab Ali, Sachidananda Behera, Pushkar Shivam, Pradeep Das, Diwakar Singh Dinesh

**Affiliations:** 1 Department of Vector Biology & Control, Rajendra Memorial Research Institute of Medical Sciences (ICMR), Agam Kuan, Patna, 800007 India; 2 Laboratory of Molecular Biochemistry and Cell Biology, Department of Biochemistry, Rajendra Memorial Research Institute of Medical Sciences (ICMR), Agam Kuan, Patna, 800007 India; 3 Department of Microbiology, Rajendra Memorial Research Institute of Medical Sciences (ICMR), Agam Kuan, Patna, 800007 India; 4 Department of Molecular Biology, Rajendra Memorial Research Institute of Medical Sciences (ICMR), Agam Kuan, Patna, 800007 India

**Keywords:** Parasite biology, Parasitic infection

## Abstract

Several Glutathione S-transferases (GSTs) enzymes, in insects, have previously been implicated in resistance developed against DDT and other insecticides. The GST enzyme particularly sigma class have important physiological role in detoxification of lipid peroxidation by-products in insects. *Phlebotomus argentipes* has been intensely exposed to DDT over years due to Indoor Residual Spray (IRS) programme for Kala-azar elimination in Bihar, India. However, in *P. argentipes*, role of GSTs in DDT resistance have not been elucidated. Here, sigma class GST of *P. argentipes* (Parg-GSTσ) was successfully cloned, expressed and purified by affinity chromatography. The recombinant Parg-GSTσ was found to be highly active towards cumene hydroperoxide and 4-HNE having specific activity 92.47 & 203.92 µM/min/mg of protein, respectively and exhibited low activity towards universal substrate CDNB *i.e*., 8.75 µM/min/mg of protein. RT-PCR and immunoblot analysis showed at least 2 and 1.8 fold overexpression of Parg-GSTσ in the single exposed and non exposed DDT resistant *P. argentipes* as compared to susceptible, implicating Parg-GSTσ also involved in DDT resistance probably by imparting enhanced stress tolerance. The DDT, H_2_O_2_ and temperature induction assays demonstrated stress-dependent induction of Parg-GSTσ expression indicating its important role in oxidative stress redressal.

## Introduction

Leishmaniasis is one of the seven neglected tropical diseases identified by World Health Organization, exhibiting broad spectrum clinical manifestations with fatal outcome^1^. Approximately, 1.5 to 2 million new cases are reported each year and causes 70,000 deaths per year with an estimated population of 350 million at risk^2^. Visceral Leishmaniasis (VL) is the most severe form of leishmaniasis and it is estimated that more than 90% cases of VL occurs mainly in five countries: India, Bangladesh, Nepal, Sudan, and Brazil^3^. According to a recent report^4^, India hosts 5634 annual cases of VL and 1916 cases of Post-Kala-Azar Dermal Leishmaniasis (PKDL) which suggests that control of VL is still a major issue. *Phlebotomus argentipes* (sand fly) is an established vector of Kala-azar or VL in Indian subcontinent. In India, northeastern regions of Bihar and it’s adjacent states like Jharkhand, West Bengal & Uttar Pradesh are predominantly affected by this disease and were targeted for VL elimination by 2017^5^. Among the various factors perceived to be involved in lack of achieving the elimination target, development of insecticide resistance may be one of the prominent factors potentially capable of jeopardizing the elimination program. Insecticides are one of the major tools to control the vectors responsible for transmission of diseases in the world. However, prolong use of insecticides may lead to development of insecticide resistance and severely impede Kala-azar elimination program. Although alternative strategies like impregnated nets, environmental management etc. are also implemented for the control of vector density, the use of insecticides has most profound impact in the elimination of vector-borne diseases. DDT was the first synthetic organochlorine insecticide to be discovered during the World War II and was extensively used all over the world in Malaria control program. Earlier, it showed quick knock down efficiency on insect vectors and longer residual effect up to 6 months (WHO Report 1997;http://www.who.int/water_sanitation_health/resources/vector357to384.pdf). However, its knock down efficiency has gradually reduced towards *P. argentipes* due to development of DDT resistance. In India, DDT was first employed in malaria control program launched in 1953 (WHO Report 1997; http://www.who.int/water_sanitation_health/resources/ vector 357to384.pdf) and was later adopted in Kala-azar elimination program^5^. However, despite continuous use of DDT in Indoor Residual Spray (IRS), Kala-azar is still prevalent in several districts of Bihar^5,6^. The failure in achieving Kala-azar elimination target 2017 in India may be due to irregularities in quality and frequency of DDT IRS^7^. Moreover, the same factor may be also implicated for gradual development of insecticide resistance^8^. DDT resistance not only impaired the IRS program but has also raised the possibility of resistance, phenotypic & genotypic fixation in the sand flies. The phenotypic fixation may be seen in the form of tolerance developed against recently introduced Pyrethroid α-Cypermethrin, whereas, genotypic fixation as knock-down resistance (Kdr) was reported in DDT resistant *P. argentipes* from Bihar^9^.

DDT resistance has been reported in many insects and more than one mechanism could be involved in acquisition of DDT resistance as reported previously in different studies on many insects^10–12^. Metabolic resistance caused by detoxifying enzymes has been widely accepted and well characterized in various insects including *Drosophila* and mosquitoes^13–15^. Among the various detoxifying enzymes involved in metabolic resistance, several classes of Glutathione S-transferase (GSTs; E.C.2.5.1.18) have been extensively explored in mosquitoes and were shown to mediate resistance by direct detoxification of DDT using GSH as cofactor^12,16^. GSTs are the major detoxification enzymes found in most organisms including insects. In vertebrates and non-vertebrates, GSTs are mainly involved in detoxification of xenobiotics, protection from oxidative damage by eliminating the by-products of oxidative stress^17^, intracellular transport of hormones and various endogenous metabolites^18^. The involvement of GSTs in insecticide resistance has been reported for organophosphates in house flies^19^, whereas, in many DDT resistant insects, including *P. argentipes*^20^, *Anopheles gambiae*^21^, *Anopheles ageypti*^22^, higher GSTs activity has been reported. Seven classes of GST are found in insects out of which only three classes, *viz* sigma^23,24^, delta^25^, and epsilon^26^ have been shown to be involved in insecticide resistance. The delta and epsilon classes of GST metabolize DDT by dehydrochlorination and thus impart DDT resistance in insects (mosquitoes)^27^. However, sigma class GST has higher affinity to organophosphorus insecticide (Fenitrothion) and play important role in oxidative stress response in fall webworm^23,24^. However, till date, no reports are available regarding the characterization of any class of GST in DDT resistance in sand flies.

The induction of stress due to herbicides and insecticides exposure is well characterized and has been reported in many insect species^28–30^. Insect GSTs may contribute in defense against oxidative damage by detoxifying or scavenging the secondary product generated by ROS or by directly metabolizing 4-Hydroxy-nonenal (4-HNE), one of the end products of lipid peroxidation (LPO), through conjugation^31^. The physiological role of GSTs as antioxidant in response to oxidative stress was reported in several insects species like, *Anopheles* mosquitoes and Honey bee^32–34^. The total GST activity was also found to be elevated in DDT resistant *Anopheles arabiensis* and *Anopheles funestus* strains to cope with oxidative stress^35,36^. Although, biochemical method for estimation and comparison of the activity of detoxifying enzymes including GSTs enzymes has been reported for insects including *P. argentipes*^37,38^ but this method cannot differentially elucidate or indicate the specific class of GST enzymes. Therefore, elucidating the role of specific class of GSTs is very crucial to understand the mechanism of detoxification and designing novel inhibitors for controlling *P. argentipes* population. The unavailability of genome database of *P. argentipes* poses a challenging task to pursue studies on specific genes including different classes of GSTs. In this study, we successfully cloned, expressed and purified the sigma class GST protein of *P. argentipes* and investigated its enzymatic properties and potential role in imparting DDT resistance in *P. argentipes*. Finally, we explored the potential role of sigma class GST as a diagnostic marker for differentiating DDT resistant and susceptible *P. argentipes* by utilizing the polyclonal antibodies raised against this purified protein and developing a modified 3,3′-diaminobenzidine (DAB) based assay^39^ to detect GST expression even in a single *P. argentipes*.

## Experimental Procedures

### Chemical and reagents

1-chloro-2,4-dintrobenzene (CDNB), Anti-DNPH antibody, Reduced glutathione (GSH), Ethacrynic acid, Hydroxynonenal (4-HNE), Glutathione reductase, Dichlorodiphenyltrichloroethane (DDT) and Dichlorodiphenyldichloroethylene (DDE) were purchased from Sigma-Aldrich (USA). All other reagents used were of analytical grades.

### Animal ethical statement

Female Balb/c mice were used for raising polyclonal antibody against purified, recombinant sigma GST protein and procedure used was reviewed and approved by Institutional Animal Ethical Committee (IAEC), Rajendra Memorial Research Institute of Medical Sciences, Patna, Indian Council of Medical Research, Government of India, New Delhi held on 14^th^ July 2017 vide no: INT-109/14.07.2017. All the methods used in the study were carried out according to the regulations and guidelines of the IAEC committee governed by CPCSEA, New Delhi, Government of India.

### Tube-Bioassay test

DDT resistant *P. argentipes* is abundantly available in the adjoining areas of the study site and, therefore, raising a resistant colony is not much troublesome; because it has developed DDT resistance to various extents in almost all the endemic districts of Bihar covered under the DDT IRS program as reported previously^40^. In contrast, there is no report of 100% susceptible *P. argentipes* field isolates from India in the recent years. To find susceptible *P. argentipes*, the Kaler village of Gaya district was chosen because in Kaler region, DDT or other insecticide has not been used in IRS for at least more than 5 years as per the information gathered from local villagers. So, we expected a higher probability of getting pure susceptible *P. argentipes* in Kaler.

Tube bioassay is the primary assay recommended by WHO for determining phenotypic resistance in insects^34,38^ including *P. argentipes*^41,42^. So, to access the susceptibility of Kaler *P. argentipes*, tube bioassay was conducted. Briefly, Kaler sandflies were collected through mechanical aspirator and field samples of female *P. argentipes* were segregated into 5 tubes of 20 sandflies each (5 replicates) and each tube was subjected to DDT exposure by confining them inside the tube containing 4% DDT impregnated paper for 1 hr exposure. After exposure period, each tube of sandflies was transferred to recovery tubes containing DDT control paper and recovery was assessed after 24 hrs. The knockdown percentage was calculated and plotted against the resistant sandflies subjected to identical tube bioassay. All sandflies (n = 100) died within 24 hrs. So, next time, we collected sandflies by mechanical aspirator from the same area for raising susceptible colony, and directly confined them inside Hilton pots for further generation of susceptible colony in our laboratory. In every generation susceptibility of sandflies was accessed through tube bioassay.

DDT has been replaced by α-cypermethrin, a pyrethroid insecticide, for IRS program in the endemic regions due to emergence of DDT resistance. To keep the samples unbiased and essentially not intermingled with any insecticide other than DDT, Vikram village of Patna district was chosen because there was no previous history of IRS with any other insecticides except DDT in this village. To obtain DDT resistant sandfly, wild *P. argentipes* were collected from Vikram village in early morning (6 A.M. to 8 A.M.) with mechanical aspirator from April-June 2014 and subjected to identical tube bioassay as described above for susceptible sandflies. We observed that >60% sandflies survived after tube assay. For raising DDT resistant colony, the Vikram sandflies surviving after tube bioassay were reared in the laboratory. The 100% pure DDT resistant sandflies were generated by exposing every generation with 4% DDT and individually confining the live female *P. argentipes* in separate Hilton pots to get the pure colony of DDT resistant.

### PCR amplification, cloning and expression of recombinant GST sigma gene (GSTσ)

The *P. argentipes* GSTσ gene specific primers were designed using *P. papatasi* GSTσ gene sequence as template. The primers used for PCR amplification were as follows: forward primer 5′-TGCTA**GGATCC***ATG*CCCAACTACAAAGTGATC-3′ and reverse primer 5′- TAGGT**AAGCTT***TTA*AACTTCAGTAACAGGTCG-3′ with a BamH1 & Hind III restriction sites (depicted in bold letters) in forward and reverse primer, respectively. Total RNA was isolated from the pool of 15 female DDT resistant sandflies using Trizol reagent according to manufacturer’s protocol. The cDNA was synthesized using cDNA synthesis Kit (Thermo-Fisher) according to manufacturer’s protocol. The GSTσ gene was PCR amplified from cDNA using Taq DNA polymerase and GSTσ gene specific primers. The PCR condition were 94 °C for 6 min followed by 35 cycles of 94 °C for 30 s, 55 °C for 35 s, 72 °C for 40 s and final extension at 72 °C for 7 min. The 612 bp PCR product was purified from 1% agarose gel using PCR product purification kit (Promega) and sequenced commercially (Xcelris, Ahmedabad, India). The obtained sequence was searched for homologous sequences using online BLAST tool and subjected to sequence alignment using Clustal W. For cloning, the PCR product was digested using restriction enzymes BamH1 and Hind III (Promega) as per the manufacturer’s protocol. The purified digested product was then ligated into digested pET-28a expression/cloning vector using Ligation Kit (Promega) and transformed in *E. coli* DH5α cells. The positive clones were purified using Plasmid purification Kit (Promega) and the insert was sequenced with T7 universal primer for pET-28a vector. For expression of recombinant GSTσ protein, the positive clone was transformed into *E. coli* BL21 (DE3) cells and expression was induced with 1.0 mM IPTG at 30 °C for 6 hrs.

### Purification of recombinant *P.argentipes* GSTσ (rParg-GSTσ)

The induced GSTσ in *E. coli* BL-21 cells were harvested at 8000 x g for 8 min and pellet corresponding to 50 ml culture was resuspended in bacterial lysis solution (50 mM Tris-Cl, 300 mM NaCl, 0.1% Triton X-100, and 10% Glycerol) supplemented with 100 µg/ml lysozyme and 1 mM PMSF. The cells were incubated for 20 min at 4 °C and 20 min at room temperature before sonicating the sample on ice. After centrifugation at 20,000 rpm for 20 min at 4 °C, the supernatant was collected and used for purification of recombinant rParg-GSTσ using Ni^+2^-NTA affinity chromatography (Thermo-Fisher) according to manufacturer’s protocol. The elution of protein was performed with 100 mM, 200 mM, 300 mM, 400 mM and 500 mM imidazole gradient prepared in bacterial lysis buffer. The purified protein was analyzed on 12% SDS-PAGE and pure homogeneous fractions were pooled and dialyzed overnight at 4 °C in Phosphate buffer saline (pH-6.5) using dialysis membrane (Thermo-Fisher). The dialyzed protein was concentrated using 15 ml 10 MWK concentrator (Merck) and protein concentration was estimated spectrophotometrically at 595 nm using Bradford reagent (Sigma-Aldrich) following manufacturer’s protocol; BSA was used as protein standard. The concentrated protein was used for performing enzyme assays and polyclonal antibody generation.

### Enzyme assays

#### CDNB assay

GSTs help in cellular detoxification of xenobiotics by catalyzing the conjugation of reduced glutathione with electrophilic xenobiotics. CDNB is the universal electrophilic substrate of all classes of GST, although, each class may differ in their affinity or specific activity towards CDNB. Thus, to determine the enzyme characteristics of purified rParg-GSTσ protein against CDNB, CDNB assay was performed as described previously^23^. Briefly, 10 µl of rParg-GSTσ protein (4 µg) was added to a mixture containing 1 mM GSH and different concentrations of CDNB (0.07–8 mM) in Sodium phosphate buffer (pH-6.5) at room temperature and formation of GSH-CDNB conjugate was monitored spectrophotometrically by measuring the increase in absorbance at 340 nm for 5 minutes. Further, similar reactions with 1 mM CDNB and different concentrations of GSH (0.01–4.0 mM) were also performed. Michaelis-Menton equation and Lineweaver-Burk plot was used to deduce the specific activity (µmol/min/mg of protein), V_max_ and K_m_ values of rParg-GSTσ protein against CDNB and GSH.

#### Optimal pH determination

To determine optimum pH for rParg-GSTσ activity against CDNB, buffers of different pH ranging from pH 5.5 to 11 were freshly prepared and used for CDNB assay. CDNB assay was performed as described above except that specific buffers of different pH were used. Precisely, the following mixed buffers were used: 50 mM 2-(N-morpholino)ethanesulfonic acid/NaOH for pH 5.5, 6.0 and 6.5; HEPES/NaOH for pH 7.0, 7.5 and 8.0; N-[tris-9hydroxymethyl)-aminomethane for pH 8.5 and 9.0; and 3-(cyclohexylamino)-1-propanesulfonic acid for pH 9.7, 10.0 and 11.0 as described previously^43^. The observed activities of rParg-GSTσ with CDNB at different pH were plotted against respective pH for determining the pH optima.

#### Peroxidase activity and 4-HNE assay

4-HNE is an intermediate product of lipid peroxidation, and very toxic to cells in high amount. 4-HNE is not produced from direct DDT metabolism, rather produced during the lipid peroxidation, which is generally aggravated by increase in stress. Apart from detoxifying electrophilic xenobiotics, GST can also participate in glutathione-mediated detoxification (peroxidation) of reactive hydroxyl radicals and 4-HNE. Thus, to explore the potential role of rParg-GSTσ protein in mediating similar functions in sand fly during oxidative stress, peroxidase and 4-HNE activities of purified rParg-GSTσ protein were investigated. Briefly, 4 µg purified rParg-GSTσ protein was added to a mixture containing 1 mM GSH and different concentrations of 4-HNE (10–100 µM) in sodium phosphate buffer and formation of GSH-4-HNE adduct was monitored spectrophotometrically by measuring increase in absorbance at 224 nm for 5 min^44,45^. For peroxidase activity assay, 4 µg purified rParg-GSTσ protein was added to a mixture containing 0.2 mM NADPH, 1 U glutathione reductase, 1 mM GSH, 1 mM EDTA and different concentrations of cumene hydroperoxide (0.08–1.0 mM) were assayed in sodium phosphate buffer. Peroxidase activity of GST was monitored by measuring the decrease in absorbance at 340 nm for 5 minutes^10,21,46^.

#### Enzyme thermostability assay

The thermostability of GSTσ may have implications for heat stress tolerance capacity of sand fly, especially in high temperature conditions. Thus, the thermostability of rParg-GSTσ was evaluated by modifying the incubation temperature of CDNB assay. Precisely, rParg-GSTσ protein was mixed with CDNB assay reaction mixture and incubated at four different temperatures *viz*. 20 °C, 35 °C, 45 °C and 55 °C for 30 minutes along with blank control (CDNB reaction mixture without protein). The temperature range was chosen based on the annual temperature variations observed in the sand fly habitat of our study site. The activity was plotted as ΔAbs 340 nm/min/mg protein through Graph Pad Prism software.

#### Dehydrochlorinase activity

Dehydrochlorination of toxic DDT insecticide yields non-toxic metabolite DDE and this conversion has been reported to be mediated by some classes of GST in insects with relevance to DDT resistance^27,47^. Thus, to delineate the role of sigma class GST protein in DDT to DDE conversion in sand fly, dehydrochlorinase activity of rParg-GSTσ was assessed according to the protocol described previously^48^. Briefly, 4.0 µg rParg-GSTσ was incubated with different concentrations of DDT (0–200 µg/ml, dissolved in methanol) in 1 ml reaction mixtures containing 20 mM sodium phosphate buffer (pH 7.6), 20 mM NaCl and 2 mM GSH, at 28 °C for 2 hrs. In parallel, control reaction comprising the same reaction mixture without insecticide was also set up in triplicate. After incubation, the reaction mixtures were extracted twice with chloroform and evaporated under gentle stream of nitrogen gas until dry. The dried residue was dissolved in 300 µl methanol and run on HPLC on a reverse-phase C18 column (Agilent ZORBAX 300SB-C18) at 23–25 °C. A mixture of acetonitrile/water 90:10 was used as mobile phase at a flow rate of 1.0 ml min^−1^. Peaks were detected at 232 nm with the ultimate 3000 UV detector (Dionex) and analyzed with Dionex Chromeleon Software. The quantity of DDT and DDE were calculated using a standard curve prepared by running known concentrations of DDT & DDE on HPLC under identical conditions. The DDT resistant sand fly homogenate was used to investigate dehydrochlorination activity in *P. argentipes*.

### Quantitative RT-PCR for Parg-GSTσ

Total RNA was isolated from laboratory reared single *P. argentipes* DDT exposed and non-exposed resistant and susceptible *P. argentipes* by using Qiagen total RNA isolation Kit. The cDNA was synthesized from 1.0 µg of isolated RNA using RevertAid First Strand cDNA Synthesis kit (Thermo Fisher) through random hexamer primers. Primers for real-time PCR were designed using the Parg-GSTσ gene sequence deduced from this study. The primers used were forward 5′-CACTGAGAAATATCCCAACCTCA-3′ and reverse 5′-TTAAACTTCAGTAACAGGTCGTTTC-3′. Real-Time PCR reaction was performed on a Qiagen multiplex RT-PCR and the relative expression and fold change was calculated according to the 2^-ΔΔCT^ method, incorporating PCR efficiency^49^ after normalization with the housekeeping gene 28 s rRNA.

### Generation of polyclonal antibody

For identification and relative quantification of Parg-GSTσ protein in sand fly lysates for immunoblot studies, polyclonal antibody against rParg-GSTσ protein was raised in mice as described previously^50^ with slight modification. rParg-GSTσ protein 60 µg was mixed with Freund’s adjuvant and injected in the peritoneal space of 4 mice. Subsequently, two booster doses with Freund’s incomplete adjuvant were administered to each mouse at two weeks interval. Two weeks after the final immunization, antibodies titre was checked by ELISA, and then the mice were sacrificed and antisera were separated from the collected blood. The antisera were incubated with immobilized *E. coli* lysate (pierce) for 2 hrs at 4 °C on a shaker. After incubation, the samples were centrifuged, anti-sera aspirated and stored at 4 °C in small aliquots.

### Immunoblotting for detection of GSTσ in single *P. argentipes*

Single *P. argentipes* female was macerated in 30 µl of modified RIPA buffer using a hand-held homogenizer. The completely homogenized mixture was then centrifuged for 25 min at 13000 rpm at 4 °C and supernatant was collected and stored in autoclaved 1.5 ml vials. Protein concentration was estimated by Bradford assay as mentioned earlier and equal amount of protein (0.7 µg) of each sand fly was resolved on 10% SDS-PAGE and immunoblotted on to a nitrocellulose membrane (0.4 µm) using semidry Trans blot apparatus (Bio-Rad). The membrane was probed with primary anti-rParg-GSTσ polyclonal antibody (1:2000) raised in mice and HRP conjugated rabbit anti-mice IgG (GeNie, 1:2000) as secondary antibody. The blot was developed with DAB/H_2_O_2_ solution (Bio-RAD) using 0.1 M imidazole as colour enhancer^51,52^. Image acquisition was performed on gel documentation machine (Gel Doc XR+, Bio-RAD).

### Stress induction assay

Stress-induced elevation in GST protein expression plays important role in stress redressal and survival of insects in the hostile environmental conditions including pesticide exposure. So, the modulation of Parg-GSTσ protein expression in sand flies exposed to different stress conditions *viz*. different temperatures (temperature stress), H_2_O_2_ (oxidative stress) and DDT (pesticide stress), were investigated through immuno-blot assays. For each assay, 6 freshly emerged female adult *P. argentipes* from each group were used in triplicate. For temperature stress assay, DDT resistant female *P. argentipes* (n = 6) were transferred to Hilton pots & pre-equilibrated at three temperatures *viz*. 4 °C, 37 °C and 55 °C and incubated for further 25 min in order to avoid their early deaths at 4 °C in BOD incubators. The time duration of 25 min was chosen only to collect live *P. argentipes* from 4 °C treatment group. After exposure, live *P. argentipes* individual samples were immediately processed for protein extraction and immunoblotting analysis. For H_2_O_2_ stress experiment, DDT resistant female *P. argentipes* were released in separate Hilton pots containing sterile cotton balls hanging from the lid and soaked with 30% H_2_O_2;_ and then left for 60 min along with control Hilton pot containing cotton ball soaked with autoclaved H_2_O only. After 60 min, total protein was immediately extracted from each female *P. argentipes* at 4 °C and immunoblot analysis was done as above. For DDT stress tolerance study, the DDT resistant female *P. argentipes* were exposed to DDT for 30 & 60 min in Bio-assay tube along with unexposed control tubes having organochlorine control. For comparative evaluation of differential Parg-GSTσ protein expression in DDT exposed and unexposed susceptible vs DDT resistant *P. argentipes*, similar bioassay procedure was followed except that the exposure time in Bio-assay tube was fixed at 40 min to allow collection of only live *P. argentipes* from susceptible colony. The total protein was extracted individually from each female *P. argentipes* (n = 4) of all groups and immunoblot analysis was performed as mentioned earlier^52^. For quantification of GSTσ band, the densitometry analysis of blots was done using ImageJ analysis software (imagej.nih.gov).

### Measurement of protein carbonylation by immunoblotting

Free oxidative radicals accumulated during oxidative stress can oxidize the amino acid residues of proteins to generate carbonyl groups^53^. Thus, protein carbonylation assay is used to comparatively evaluate the oxidative stress level. To compare the oxidative stress level generated after DDT exposure in susceptible vs resistant female *P. argentipes*, protein carbonylation assay was performed using Dinitrophenyl hydrazine (DNPH) as described previously^54^. Briefly, 6 µg total proteins was mixed with DNPH (1 mM) in 2 mM HCl and incubated for 20 min at room temperature to form carbonyl derivative dinitrophenylhydrazone. The reaction mixture was separated on a 10% SDS-PAGE and immunoblotted on to a nitrocellulose membrane. The membrane was probed with anti-DNPH antibody (Sigma-Aldrich, 1:2000) raised in rabbit and HRP conjugated goat anti-rabbit IgG (GeNie, 1:2000) as secondary antibodies. The blot was developed with DAB/H_2_O_2_ solution (Bio-RAD) by standard protocol as described earlier^55^.

### Statistical analysis

Statistical analysis was carried out using GraphPad Prism 5.0 software (GraphPad Software Inc., La Jolla, CA, USA). Student’s *t*-test was used to estimate the statistical significance of the differences between groups. Differences between groups were considered statistically significant when p value was less than 0.05.

## Results

### Establishment of 100% DDT resistant and susceptible colony of *P. argentipes* by Tube Bio-assay

**Figure 1:**
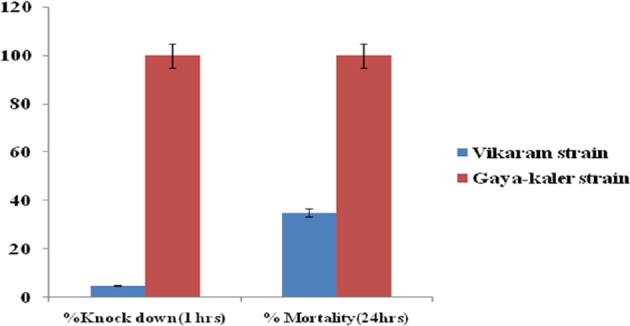
Susceptibility assay of *Phlebotomus argentipes* sandflies collected from village Vikaram (Patna) and Kaler (Gaya) was performed by Tube Bio-assay after exposure to 4% DDT. The Kaler colony showed 100% mortality within 24 hrs (Brick red bar).

Generating a resistant colony of *P. argentipes* is not much troublesome as it has developed DDT resistance to various extents in almost all the endemic districts of Bihar; and has been reported in previous study^40^. However, it is difficult to get 100% susceptible *P. argentipes* in field from India. Thus, to find DDT susceptible sandfly, we selected Kaler village of Gaya district because DDT or other insecticide was not used here in IRS for at least more than 5 years. The tube bio-assay results showed that *P. argentipes* from Kaler region is 100% susceptible to 4% DDT with complete knockdown effect observed in 60 min (Fig. 1). In contrast, the resistant colony was established from Vikaram village, Patna District, as there was no history of other insecticide spray except DDT in this area. The tube bio-assay on *P. argentipes* from Vikaram village showed 0% knockdown to 4% DDT in 60 min and exhibited more than 60% DDT resistance after 24 hrs of recovery period (Fig. 1). The 100% pure resistant population of *P. argentipes* from Vikaram was further reared at our institutional laboratory and obtained 100% resistant colony in the 7^th^ generation. Both the strains were reared separately in laboratory throughout this study.

### Amplification of Parg-GSTσ gene

**Figure 2:**
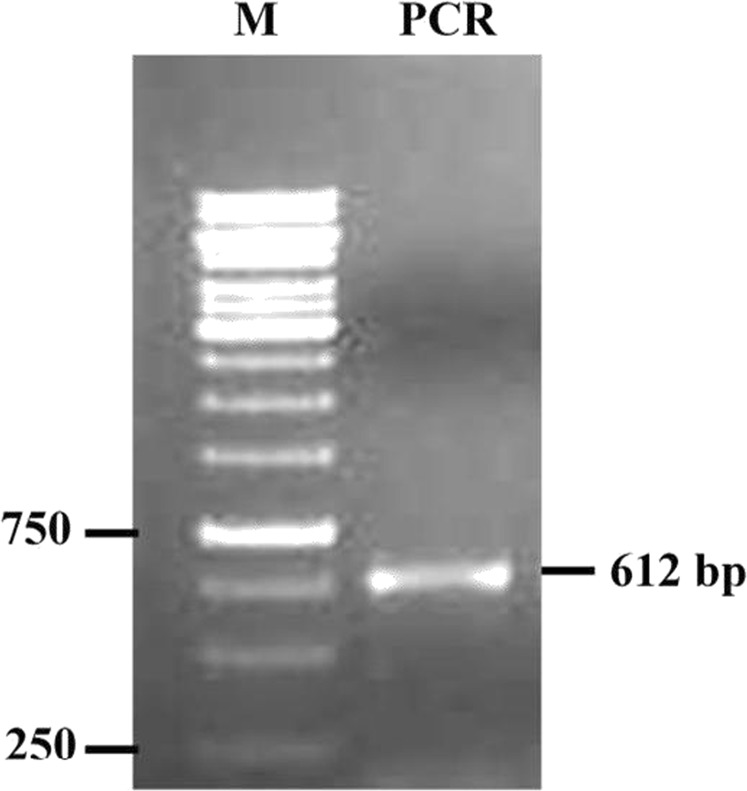
PCR product run on 1% agarose gel showing Lane 1 1.0 kb DNA marker; Lane 2 band of 612 bp corresponding to Parg-GSTσ gene.

**Figure 3:**
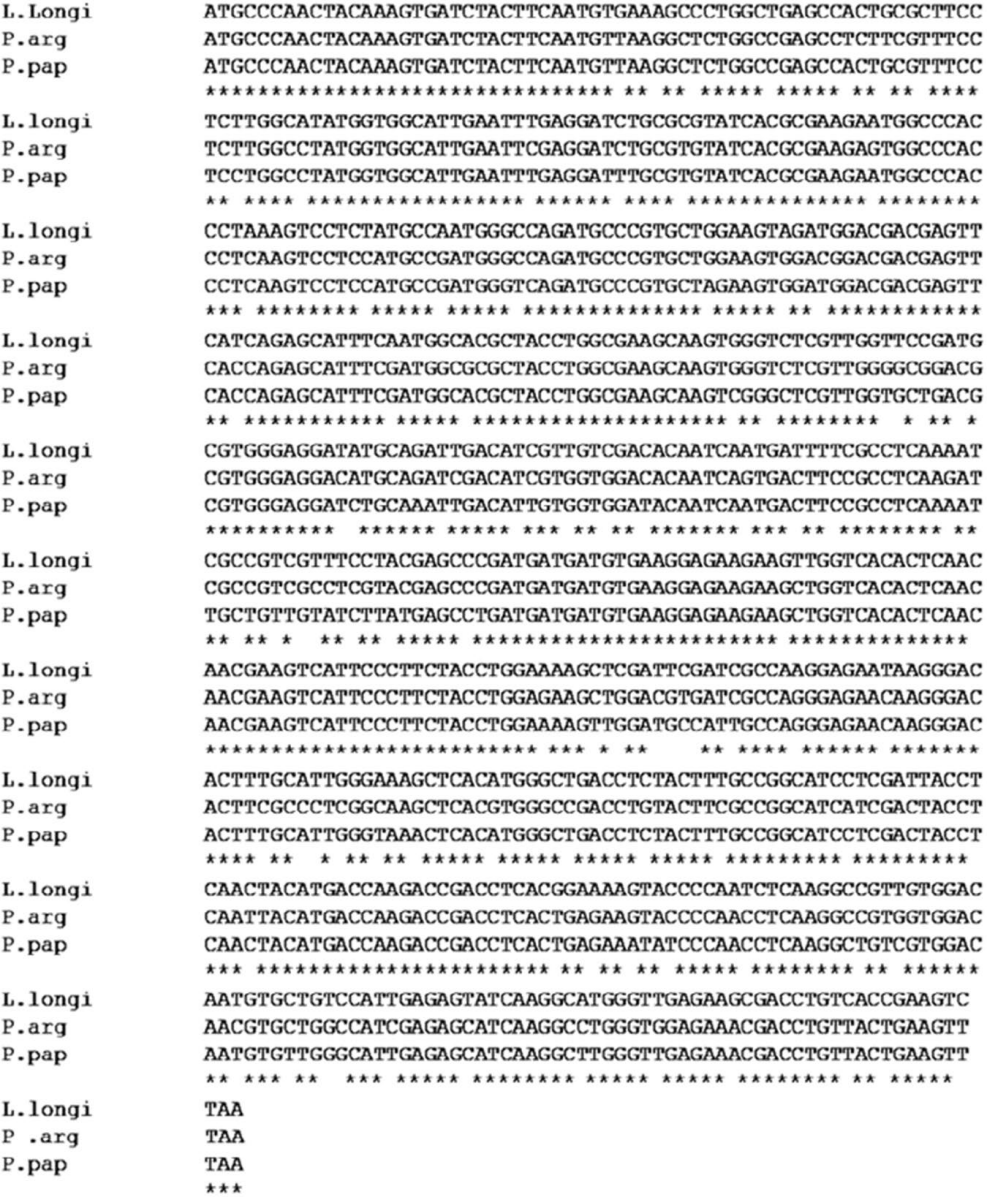
Multiple nucleotides sequences alignment of Parg-GSTσ genes by Clustal W: Nucleotides sequence alignment analysis of *P. argentipes* Parg-GSTσ gene sequence shows 91% and 82% homology with sigma class GST from *P. papatasi* & *L. longipalpis*, respectively. Identical nucleic acid residues among all species are depicted with asterisk at the base.

At the time of this study, the genome sequence of *P. argentipes* was not available and the GST-encoding gene sequence of *P. argentipes* is still uncharacterized. Thus, we used the publicly available complete cDNA sequence of GSTσ of *P. papatasi* as the template to design primers for amplification of GSTσ gene of *P. argentipes*. PCR amplification with designed primers showed a single band of ~612 bp as visualized on a 1% agarose gel stained with ethidium bromide (Fig. 2). Sequencing of 612 bp PCR product and BLAST analysis identified the amplified product as GSTσ gene. The PCR product was then cloned and sequencing of several clones revealed a single sequence of GSTσ having 91% and 81% identity with *P. papatasi* and *L. longipalpis* GSTσ gene (Fig. 3) in Blastx search and Clustal W analysis. The gene sequence was submitted to GenBank and the accession number was obtained (Accession: MG431969).

### Purification of rParg-GSTσ protein

**Figure 4:**
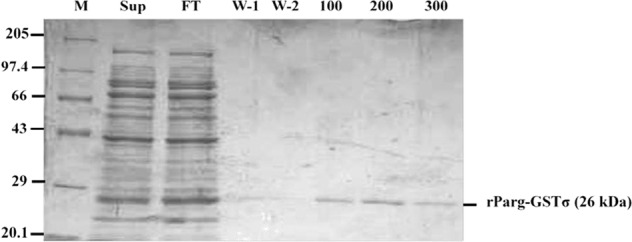
Purification of recombinant Parg-GSTσ protein using Ni-NTA column. All protein samples were processed and run on 12% SDS-PAGE to evaluate recombinant protein purification. Lane 1- Mol. Wt. Marker; Lane 2- Supernatant; Lane 3- Flow through; Lane 4 &5- Wash 1 & 2; Lane 6 to 8- elute 100, 200 & 300 mM imidazole, respectively.

To obtain rParg-GSTσ fusion protein, 612 bp PCR product of GSTσ gene was cloned in pET28a expression vector and positive clone was transformed in BL21 *E. coli* competent cells. The expression was induced with 1 mM IPTG and confirmed by western blotting with anti-His antibody (1:4000, G-Biosciences) (data not shown). The purification of rParg-GSTσ using Ni^+2^-NTA agarose beads showed a single band of ~26 kDa (including Histidine tag 3.52 kDa) in the elute fraction on 12% SDS-PAGE confirming that the rParg-GSTσ is expressed in soluble form (Fig. 4) which is considered ideal for enzyme activity assays.

### Enzyme kinetic of rParg-GSTσ proteins

#### GSH & CDNB reactivity

**Figure 5:**
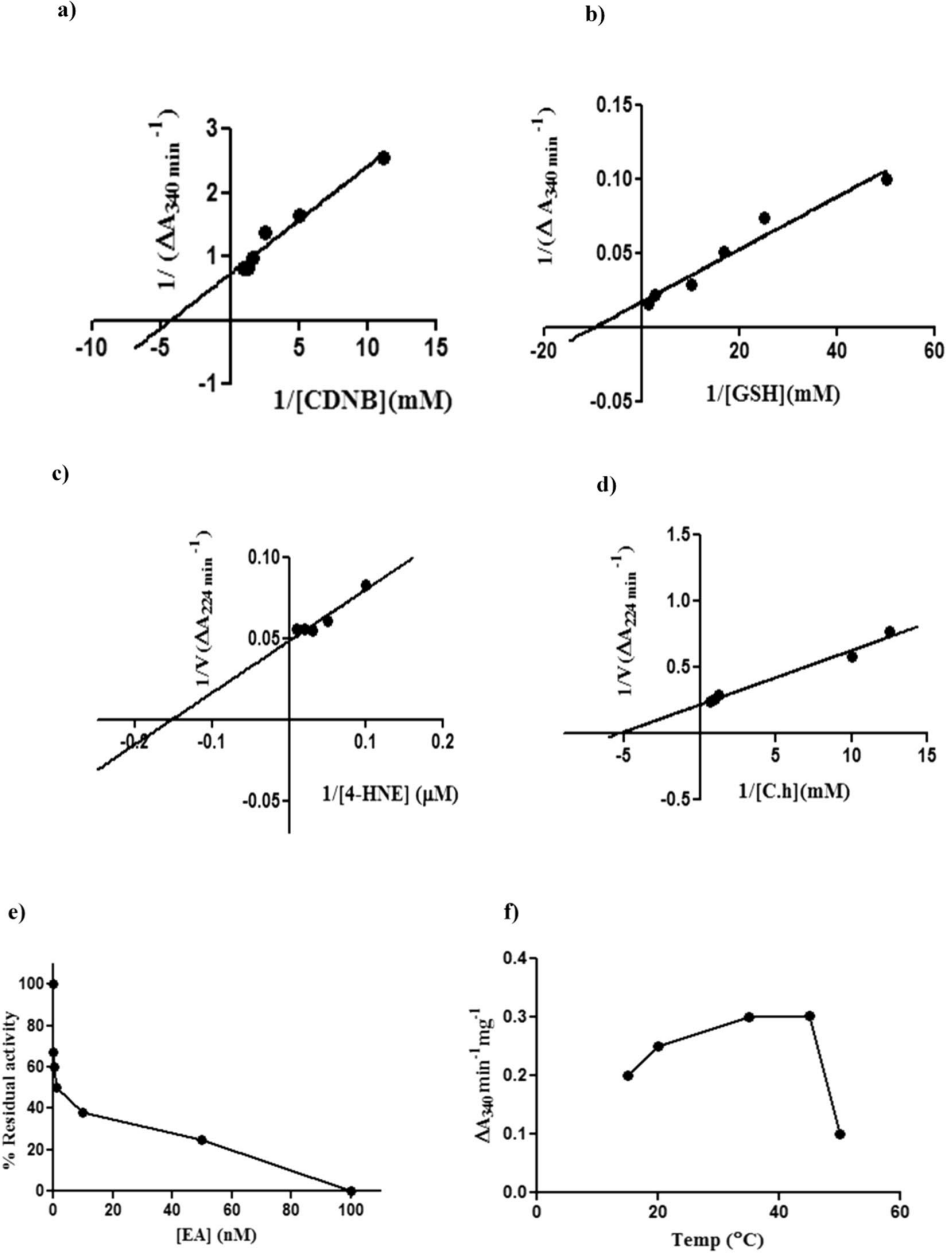
Enzyme kinetics analysis of Parg-GSTσ: (**a**) Activity of rParg-GSTσ with different concentrations of CDNB; (**b**) Activity of rParg-GSTσ with different concentrations of GSH; (**c**) rParg-GSTσ reactivity with 4-HNE; (**d**) Activity of rParg-GSTσ with different concentrations of cumene hydroperoxide shown as reciprocal plot; (**e**) % Residual activity of rParg-GSTσ with Ethacrynic acid; (**f**) rParg-GSTσ activity at different temperature.

CDNB and DCNB are the colorimetric universal substrates of GST and are also utilized in biochemical assays for the determination of GST elevation in resistant insects^38,55^. So, rParg-GSTσ was initially characterized with these substrates. The enzyme kinetic analysis was conducted with various concentrations of CDNB, DCNB and GSH (mM) as per the protocol given in Methodology. The K_m_ values of rParg-GST for CDNB & GSH were found to be 0.39 mM & 0.13 mM of protein, respectively. However, Vmax for CDNB & GSH were found to be 1.39 mM/min/mg and 68.92 mM/min/mg of protein respectively, as deduced from Michaelis-Menton and Lineweaver-Burk plot analysis in Graph Pad Prism software. The specific activity of rParg-GSTσ at equimolar concentration (0.8 mM) of CDNB and GSH (0.8 mM) was found to be 8.75 µM/min/mg & 72.5 µM/min/mg of protein, respectively (Fig. 5a,b) (Table 1).

**Table 1:** Kinetic analysis of rParg-GSTσ: V*max*, K*m* and Specific Activity (µM/min/mg) of rParg-GSTσ with different substrates (CDNB, GSH, 4-HNE & cumene hydroperoxide).

Substrate	Vmax	Km	Specific Activity (µM/min/mg)
CDNB (mM)	1.7	0.39	8.75
GSH (mM)	68.92	0.13	72.5
4-HNE (µM)	19.57	5.02	203.92
Cumene hydroperoxide (mM)	4.48	0.18	92.47

#### rParg-GSTσ reactivity against 4-HNE

4-HNE is the by-product of lipid peroxidation (LPO)^56,57^ and toxic to cells in high amount. The rParg-GSTσ was found to be highly active against 4-HNE exhibiting Km & Vmax values of 5.02 µM & 19.57 µM/min/mg of protein, respectively (Fig. 5c). The deduced specific activity of rParg-GSTσ against 4-HNE was found to be 203.92 µM/min/mg of protein (Table 1).

#### Peroxidase activity assay

Peroxidase activity is an important attribute for stress redressal caused by ROS^58^. Thus, we explored the peroxidase activity of rParg-GSTσ of *P. argentipes* against cumene hydroperoxide, and found that it has high affinity to cumene hydroperoxide exhibiting Km & Vmax values of 0.18 mM & 4.48 mM/min/mg, respectively (Fig. 5d). The deduced specific activity of rParg-GSTσ against cumene hydroperoxide was found to be 92.47 mM/min/mg of protein (Table 1). Interestingly, rParg-GSTσ exhibited high activity for cumene hydroperoxide, as compared to recombinant Delta class GSTs from *Anopheles gambiae* (e.g., AgGSTD1-5 and AgGSTD1-6)^21^ and *D. melanogaster* (e.g., DmGSTD1 and DmGSTD21)^10^. Hence, these findings suggest that Parg-GSTσ may play a role in cellular-antioxidative defence against oxidative damage induced by DDT and other xenobiotics.

#### Inhibitor assay

Ethacrynic acid (EA) is an inhibitor of GSTs and well reported in several previous studies^36^. So, to check whether EA also inhibits the Parg-GSTσ as well as to get a mechanistic insight into the inhibition reaction, kinetic study with different concentrations of EA was performed. The inhibition assay of rParg-GSTσ with EA showed decrease in residual activity with increasing concentration of EA. The IC_50_ of EA against rParg-GSTσ activity was 50 nM, whereas, complete inhibition of rParg-GSTσ activity was observed at 0.1 µM (Fig. 5e).

#### pH & Temperature

pH is an important determinant for the proper function of enzymes. So, to determine the optimum pH, rParg-GSTσ activity was assayed in buffer of different pH and substrate CDNB concentration as given in the methodology section. The optimum GST activity was observed at pH 6.5 and it decreased gradually with increase or decrease of pH. Optimum temperature is also an important parameter for enzyme activity, specially for cold blooded organisms such as *P. argentipes* and the steep variation in average temperature observed during various seasons in Eastern India (ranging from 14 °C in winter to 45 °C in summer), the correlation between GST activity and temperature may assume pertinent for disease transmission as well as insecticide resistance. Thus, the activity of rParg-GSTσ was assayed at different temperatures and the results showed that the rParg-GSTσ have max temperature range from 35–45 °C indicating its stability at higher temperature as well as perceived role in survival of sand flies during harsh temperature in summer season (Fig. 5f). Moreover, the activity of rParg-GSTσ sharply decreased at temperature beyond 45 °C which can be clearly correlated with decrease in *P. argentipes* population during the month of June-July when the average temperature is close to 45 °C.

### Induction of Parg-GSTσ in *P. argentipes* by temperature, H_2_O_2_ and DDT

**Figure 6:**
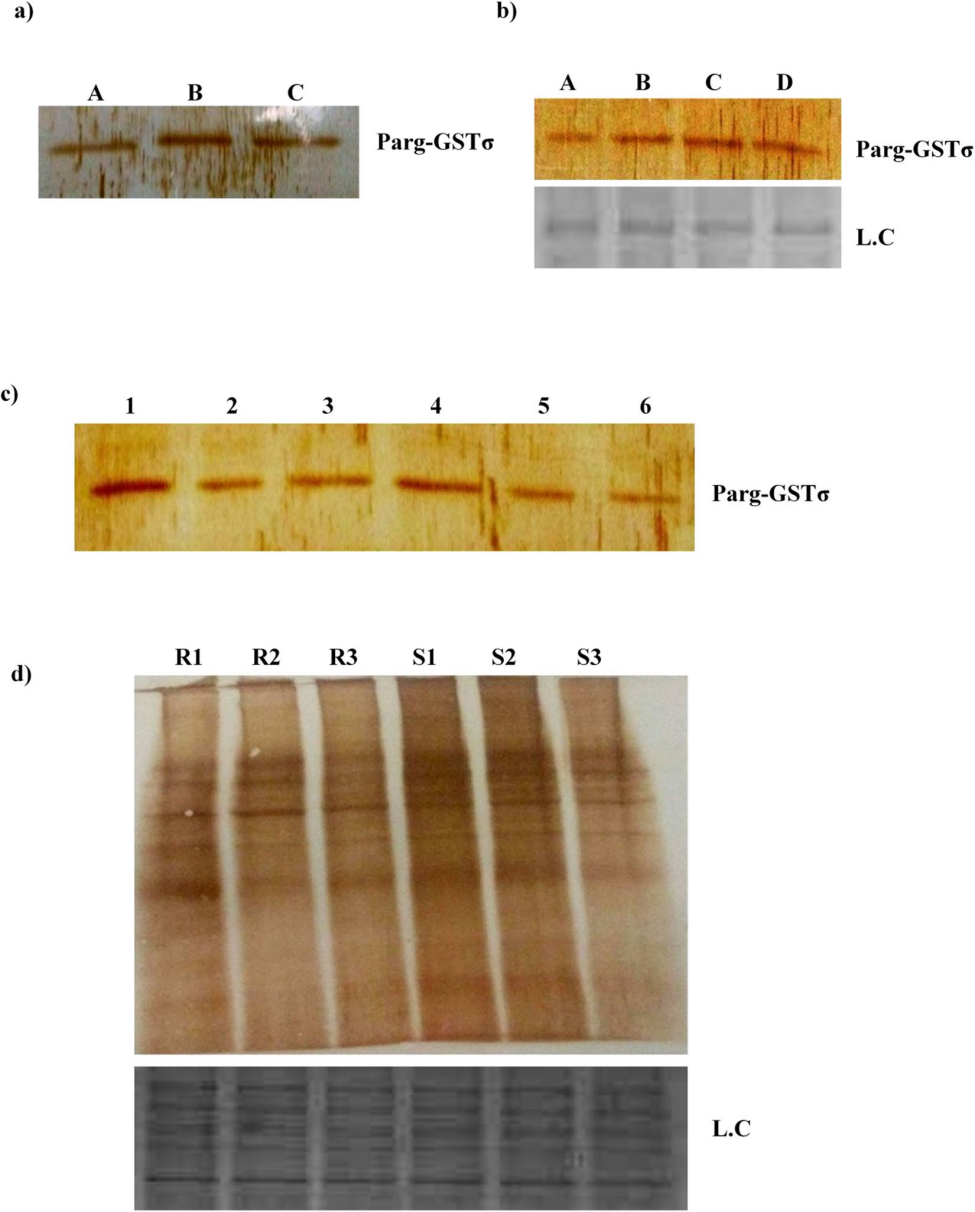
Immunoblot analysis showing: (**a**) expression of Parg-GSTσ in *P. argentipes* kept at different temperatures, Lane A: + 4 °C, Lane B: + 37 °C, Lane C: + 55 °C; (**b**) Expression of Parg-GSTσ in H_2_O_2_ non-exposed Vs exposed *P. argentipes*, Lane A & B Non exposed, Lane C & D exposed; (**c**) Expression of Parg-GSTσ in 4% DDT exposed Vs non-exposed *P. argentipes*, Lane 1 & 4 show: DDT exposed (60 min & 30 min, respectively), Lane 2 & 3 show: Non exposed *P. argentipes* (60 min), Lane 5 & 6 show: Non exposed *P. argentipes* (30 min); (**d**) Carbonylation assay of DDT exposed resistant and susceptible *P. argentipes* shows more carbonylation in susceptible sandflies from S1-S3 in upper panel and lower panel shows loading control.

GSTs play an important role in oxidative stress response^32^. The importance of stress induced defense mechanism and its role in survival of insects against pesticide have been reported in several studies^59^. Moreover, GST level was also found to be elevated in *Nilaparvata lugens*^25^ primarily to protect tissues by conferring resistance against oxidative damage. In our case, immuno-blotting of *P. argentipes* incubated at different temperatures revealed 2.3 fold higher expression of Parg-GSTσ at 37 °C and 1.9 fold lower expression at + 4 °C (Fig. 6a) indicating strong modulation of GSTσ in *P. argentipes* during temperature stress. Similarly, modulation of Parg-GSTσ expression was also observed with other stress inducers viz. H_2_O_2_ & DDT exposure. Immunoblot analysis of *P. argentipes* lysates (exposed Vs non-exposed) showed 1.9 fold & 2.0 fold higher expression of Parg-GSTσ in 30% H_2_O_2_ and 4% DDT exposed *P. argentipes*, respectively, as compared to non-exposed sandflies (Fig. 6b,c). Band intensities of all the immunoblots were normalized with coomassie stained protein bands.

Thus, our results showed a vivid and strong induction of Parg-GSTσ protein expression on stress exposure supporting a stress-dependent mechanism of regulation of Parg-GSTσ protein expression that is aptly capable of alleviating stress and may ensure survival in *P. argentipes*.

### Carbonylation assay

Proteins are the most sensitive targets of free radical species accumulated in the cells due to redox imbalance. The importance of oxidative modification of the proteins generating carbonyl groups on amino acid residues has been reported in many studies^54,60^. To check the ROS burden after DDT exposure in a single DDT resistant and susceptible *P. argentipes*, the carbonylation assay was conducted by western blotting of 4% DDT exposed resistant and susceptible colonies. The immunoblot probed with anti-DNP antibody showed at least 2.5 fold higher carbonylation of proteins in susceptible as compared to resistant colony (Fig. 6d) indicating higher ROS burden in susceptible colony after DDT exposure.

### Quantitative real time PCR of GST in single *P. argentipes*

**Figure 7:**
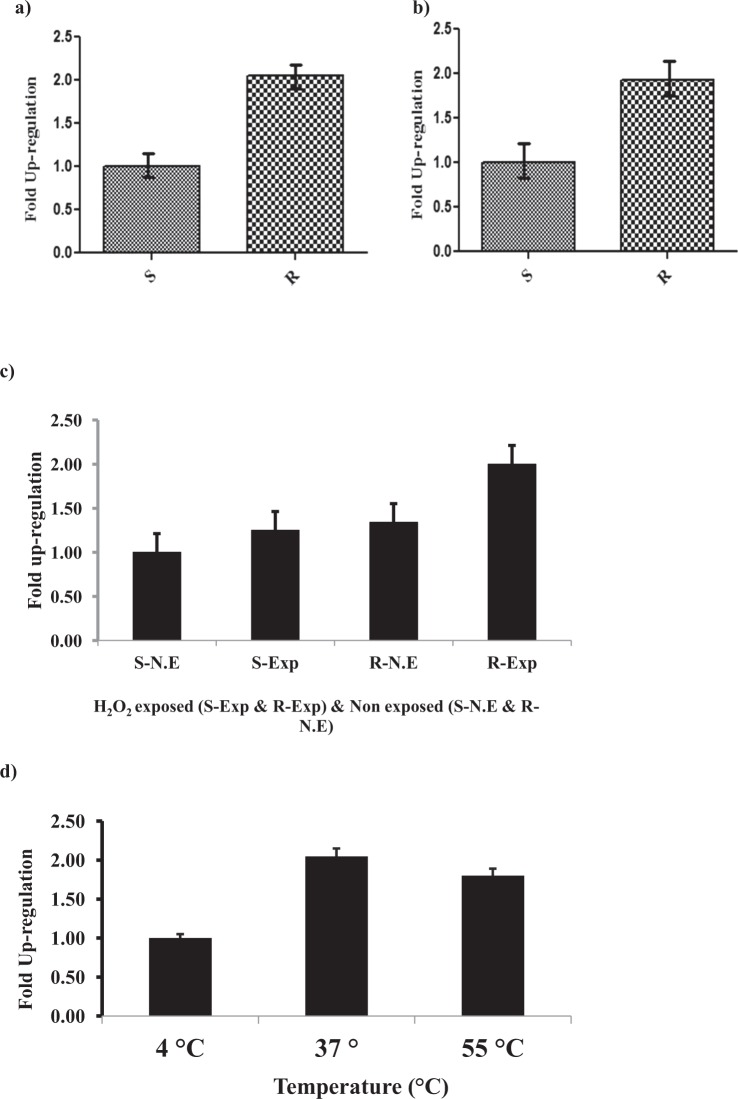
Quantitative PCR of Parg-GSTσ gene: (**a**) RT-PCR of DDT exposed susceptible (S) & resistant (R) *P. argentipes*; (**b**) RT-PCR of DDT non-exposed susceptible (S) & resistant (R) *P. argentipes*; (**c**) RT-PCR showing expression of Parg-GSTσ gene after exposure of sensitive and DDT resistant *P. argentipes* with H_2_O_2_ and (**d**) different temperatures (4 °C, 37 °C, 55 °C).

Previously, quantitative PCR has been used to implicate GST in pesticide resistance by determining elevation of GST gene expression in DDT and Permethrin resistant *Anopheles gambiae*^61^ and *Aedes Ageypti*^26^, respectively. To undermine the role of Parg-GSTσ in imparting DDT resistance to *P. argentipes*, relative expressions of Parg-GSTσ gene in DDT resistant and susceptible *P. argentipes* was evaluated by real time PCR. Real time PCR analysis from three separate experiments indicated that the expression of Parg-GSTσ was 2 ± 0.2 fold higher in DDT exposed resistant *P. argentipes* as compared to susceptible (Fig. 7a); whereas, the expression of Parg-GSTσ was found to be 1.8 ± 0.1 fold higher in non-exposed DDT resistant *P. argentipes* as compared to susceptible (Fig. 7b). On the other hand, similar upregulation of Parg-GSTσ was also observed in RT-PCR study of H_2_O_2_ and high temperature exposed sand flies. After H_2_O_2_ exposure, 1.4 ± 0.1 fold higher expression was observed in non-exposed DDT resistant and 1.6 ± 0.1 fold higher in exposed DDT resistant as compared to non-exposed and exposed susceptible *P. argentipes*, respectively (Fig. 7c). Temperature stress also induced 1.9 ± 0.2 fold higher expression of Parg-GSTσ gene at 37 °C in DDT resistant as compared to susceptible *P. argentipes*. Although, expression of Parg-GSTσ gene was also found to be modulated at 55 °C (Fig. 7d) but not all sandflies survived at higher temperature; no significant changes was observed at 4 °C. In conclusion, the results showed that DDT resistant sandflies have upregulated Parg-GSTσ gene expression under ambient conditions as compared to susceptible (Fig. 7d). Additionally, the resistant sandflies exhibited higher magnitude of induction of Parg-GSTσ gene under stress conditions as compared to susceptible colony which may impart enhanced stress tolerance to the resistance phenotype. Notably, inverse correlation was observed between Parg-GSTσ gene expression and ROS levels observed in carbonylation assay (Fig. 7d) under native as well as stress conditions.

### Detection of differential expressions of Parg-GSTσ protein in DDT resistant and susceptible *P. argentipes* through western blotting

**Figure 8:**
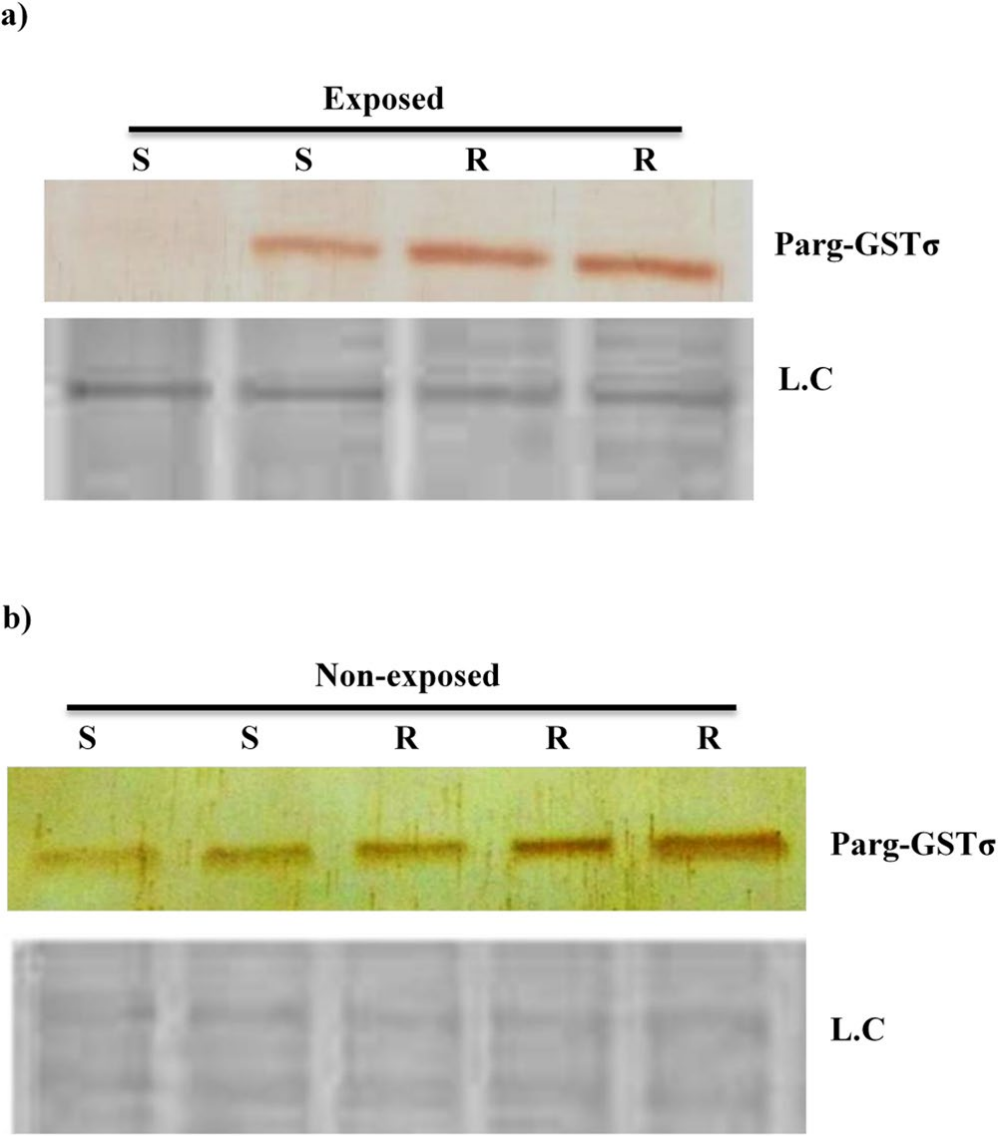
Immunoblot analysis showing: (**a**) Expression of Parg-GSTσ in DDT exposed susceptible (S) and Resistant (R) *P. argentipes* in upper panel; (**b**) Expression of Parg-GSTσ in DDT non-exposed Susceptible (S) and Resistant (R) *P. argentipes* in upper panel. L.C. represents loading control in both lower panels.

To validate the differential gene expression of Parg-GSTσ gene at translational level, a modified DAB-based assay was developed and employed to enable immunoblot detection of Parg-GSTσ protein in a single sand fly. Immunoblotting assay of single resistant & susceptible *P. argentipes* revealed 2.8 fold higher expression of Parg-GSTσ in resistant *P. argentipes* as compared to susceptible when exposed to 4% DDT (Fig. 8a), whereas the expression of Parg-GSTσ was 2 fold higher in non-exposed DDT resistant of *P. argentipes* as compared to susceptible (Fig. 8b). Thus, these results are in concordance with results of high Parg-GSTσ gene expression in DDT resistant *P. argentipes* in RT-PCR.

### Dehydrochlorinase activity

The importance of dehydrochlorinase activity in the development of DDT resistance in insects has been reported in several studies earlier^27,47^. Several classes of GST enzymes directly metabolize DDT through dehydrochlorination activity utilizing GSH as cofactor^12,16^. So, the dehydrochlorinase activity of rParg-GSTσ was determined using HPLC. The results showed that rParg-GSTσ doesn’t have dehydrochlorination property as no significant peak of DDE was observed in HPLC graph as compared to positive control (data not shown).

## Discussion

In spite of non-availability of *P. argentipes* GST gene sequence in database, we successfully cloned the sigma class GST gene and submitted its complete coding sequence (Accession no: MG431969) in the public NCBI database. The rParg-GSTσ protein was expressed, purified and characterized its enzymatic properties along with kinetic parameters. Further, we analyzed Parg-GSTσ expression profile in DDT resistant and susceptible sand fly and investigated it’s potential role in reducing oxidative damage caused by DDT. We also developed a method for detection of GST protein even in single sand fly and used it to demonstrate the differential expressions of GST in DDT resistant and susceptible *P. argentipes*. Finally, we studied the role of Parg-GSTσ in imparting stress tolerance to these sand flies which is of potential importance in acquisition of DDT resistance.

The basis for classification of GSTs into specific class is based on percentage homology; and proteins having more than 40% identity are grouped within a single class^62,63^. Our BLAST results with cloned Parg-GSTσ gene sequence showed 91% and 89% identity with *P. papatasi* and *L. longipalpis* sigma class GST (Fig. 3) confirming that the GST gene reported in this study belongs to the sigma class. Several previous studies have reported that the sigma class GSTs from vertebrates and invertebrates has low but detectable activities for universal substrate CDNB^64^. The specific activities of sigma class GST in *Drosophila* have been reported to be 0.49 µM/min/mg^64^ and 0.39 µM/min/mg of protein^65^ for CDNB substrate. However, we observed several folds higher specific activity of rParg-GSTσ sigma protein against CDNB from *P. argentipes*. The high specific activity against CDNB may reflect the adaptation gained to cope up with the continuous pressure exerted by DDT spray because GSTs are important detoxifying enzymes which are involved in xenobiotics detoxification including DDT^12,66^. However, rParg-GSTσ showed no DDT dehydrochlorination activity which reflects that its specific role is confined to cell signaling and protection against oxidative damage. Apart from that, our rParg-GSTσ activity assays performed at different pH showed that phosphate buffer pH 6.5 is the appropriate buffer which should be utilized for the measurement of sigma class GST activity in *P. argentipes* whereas, Tris-Cl buffer pH 6.5 completely inhibits the activity. Thus, our results extend support to the use of phosphate buffer, pH 6.5, in GST bio-chemical assay protocol currently recommended by WHO for the detection of insecticide resistance by measuring elevation of total GST activity in insects. Interestingly, the activity assays performed at different temperatures showed that rParg-GSTσ activity increases with increase in temperature exhibiting maximum activity around 45 °C which reflects the remarkable thermo-stability of rParg-GSTσ aptly conducive to sustain high density growth of sand flies in summer season when average maximum temperature reaches between 40 °C to 50 °C or even higher.

Peroxidase activity of GSTs are of particular importance in insects because, in contrast to Se-dependent peroxidase activity of glutathione peroxidase proteins, peroxidase activity of GSTs is Se-independent^46^; aiding in cellular anti-oxidant defense by reducing organic hydroperoxides^67^. In addition, sigma class GSTs also metabolize 4-HNE which is a by-product of LPO^68^ produced during the breakdown of long chain lipid hydroperoxides^69^. In this context, the observed activity of rParg-GSTσ towards cumene hydroperoxide and 4-HNE indicates its role in antioxidant defense and metabolism of LPO by-product in *P. argentipes*. Earlier in *D. melanogaster*, GSTσ was reported to be involved in LPO pathway through its ability to catalyze the conjugation of GSH to 4-HNE^65^. Moreover, an efficient protection against LPO by-product in sandflies may be a manifestation of higher specific activity and/or up-regulation of GST protein expression levels, either or both of which may also contribute to stress tolerance and/or insecticide resistance. The DDT resistant sandflies inherently exhibited significantly lower oxidative stress levels as compared to sensitive ones as assessed by protein carbonylation assay. The observed up-regulation of Parg-GSTσ protein level in DDT resistant sandflies as compared to sensitive ones and stress-induced induction of Parg-GSTσ protein expression levels strongly endorses our view that Parg-GSTσ has a protective role in acquisition of DDT resistance mediated by enhancing stress tolerance and/or activating stress redressal mechanism. Moreover, DDT spray may also force a shift in habitat of sand flies from inside house to outside as reported by Poche *et al*.^70^, where an increase in abundance of *P. argentipes* was observed in outside vegetation area post-IRS in Bihar. This shift in habitat or increase in outdoor density increases exposure to various xenobiotics including plant based allelochemicals, which in turn can induce GSTs as reported previously in several insects^71–75^. In *M. destructor*, the larvae exposed to allelochemicals showed elevated levels of sigma and delta GST RNA in their midgut^72^. Thus, the DDT exposure/spray may ultimately culminate into elevated GST levels in surviving sandflies and contribute in acquisition of DDT resistance and/or stress tolerance. Indeed, Oliver *et al*., has recently showed that, GST plays a pivotal role in neutralizing oxidative burden in DDT resistant *Anopheles* mosquitoes and is found to be elevated in response to oxidative damage caused by insecticide and xenobiotics^35^; which further consolidates our hypothesis.

Apart from its perceived role in stress tolerance and DDT resistance, Parg-GSTσ may serve as a valuable diagnostic tool to differentiate between sensitive and DDT resistant sand flies, aided by the observed up-regulation of Parg-GSTσ in DDT resistant sand flies. Until now, this differentiation is based on total GST biochemical assay protocol recommended by WHO for mosquitoes and extended to *P. argentipes*. However, it requires skilled hands and sophisticated instruments, often difficult to realize in real-time and on the spot at field conditions. In this study, the differential expression of Parg-GSTσ was successfully detected at single sand fly level by a modified DAB-based western blotting to differentiate between DDT resistant and sensitive sand fly utilizing polyclonal anti-rParg-GSTσ antibody raised by us. Our result showed a visually discriminating difference in Parg-GSTσ protein levels between the resistant and sensitive sand flies and thus, unveiling a method of potential diagnostic importance in differentiating between DDT resistant and sensitive sand flies in the field samples of *P. argentipes*. Previously, the GST elevation in other insects has been detected by real time PCR and ELISA^26,76^. However, as far as our knowledge, there is no report available till date for detecting GST differential expression in single sand fly or other insects by using DAB system; which has an added advantage of being easily amenable in visible range^77^ with single sand fly in near future. Apart from the diagnostic perspective, our study also unveils new potential target for designing new alternative insecticide against *P. argentipes*; a domain severely lacking in the fields of vector research in *P. argentipes*, largely impeded by the unavailability of complete genome database, and is of immense importance for developing countries hosting significant leishmaniasis disease burden and transmission vector abundance including India.
